# Loss of proton‐sensing GPR4 reduces tumor progression in mouse models of colon cancer

**DOI:** 10.1002/1878-0261.70045

**Published:** 2025-05-21

**Authors:** Leonie Perren, Moana Busch, Pedro A. Ruiz, Ermanno Malagola, Valeria Baumeler, Federica Foti, Adelina Gross, Tobias Grütter, Hendrik Edel, Cordelia Schuler, Kristina Handler, Glenn De Lange, Isabelle C. Arnold, Cheryl de Vallière, Klaus Seuwen, Martin Hausmann, Gerhard Rogler

**Affiliations:** ^1^ Department of Gastroenterology and Hepatology University Hospital of Zurich, University of Zurich Zurich Switzerland; ^2^ Institute of Experimental Immunology University of Zurich Zurich Switzerland; ^3^ Comprehensive Cancer Center Zurich Zurich Switzerland

**Keywords:** colorectal cancer, GPR4, inflammatory bowel disease, pH‐sensing G protein‐coupled receptors

## Abstract

We aimed to understand the role of G protein‐coupled receptor 4 (GPR4) in tumorigenesis. GPR4 is a pH‐sensing receptor that is activated by acidic extracellular pH. GPR4 is expressed primarily in vascular endothelial cells (ECs). Intestinal tissue from patients with inflammatory bowel disease (IBD) shows increased expression of GPR4. Patients with IBD have a significantly increased risk of developing colorectal cancer (CRC). In the MC38 model, *Gpr4*‐deficient mice showed significantly reduced tumor size and weight compared to wild‐type (WT) mice. This effect correlated with a significant increase in IL2 protein and natural killer (NK)1.1^+^ cells in tumor tissue in *Gpr4* ^−/−^ compared to WT. In the azoxymethane (AOM)/dextran sodium sulfate (DSS) model of CRC, *Gpr4*‐deficient mice showed significantly reduced tumor progression and number of apurinic/apyrimidinic (AP) sites. *Gpr4‐*deficient mice showed a significantly increased number of NKp46^+^ cells in tumor tissue, and increased numbers of NK cells were confirmed by qPCR and flow cytometry. The absence of GPR4 significantly attenuated tumor progression in the colon of mice, and this result correlated with increased cytotoxic cell activity and reduced presence of tumor‐associated macrophages and neutrophils. GPR4 represents a potential new target for therapeutic intervention.

## Graphical abstract

G protein‐coupled receptor 4 (GPR4) is a pH‐sensing receptor activated by acidic pH. GPR4 expression is increased in patients with inflammatory bowel disease who are at high risk of developing colorectal cancer. In mouse models, loss of GPR4 attenuated tumor progression. This correlated with increased IL2 and natural killer cell activity. GPR4 represents a new target for therapeutic intervention.

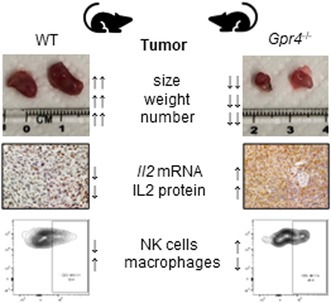

**Abbreviations**
AOM: azoxymethane
AP: apurinic/apyrimidinic
cAMP: cyclic adenosine monophosphate
CD: Crohn's disease
cDNA: complementary DNA
DAB: 3,3′‐diaminobenzidine
DSS: dextran sodium sulphate
ECs: endothelial cells
FSC: forward scatter
*Gapdh*
: glyceraldehyde‐3‐phosphate dehydrogenase
GFP: green fluorescent protein
GPR4: G‐protein‐coupled receptor 4
GPRs: G‐protein‐coupled receptors
HBSS: Hank's balanced salt solution
HE: hematoxylin and eosin
IBD: inflammatory bowel disease
IHC: immunohistochemistry
IL: interleukin
*Klrb1c*
: killer cell lectin‐like receptor subfamily B, member 1
NK: natural killer
PBS: phosphate‐buffered solution
qPCR: real‐time quantitative polymerase chain reaction
*s.c*.: subcutaneous
SD: standard deviation
SEM: standard error of the mean
SSC: side scatter
UC: ulcerative colitis
WT: wild‐type

## 1 Introduction

GPR4 is a pH‐sensing receptor that is activated by an extracellular pH below the physiologic level of pH 7.4 [1]. GPR4 senses extracellular protons via histidine residues located in the extracellular region of the receptor, leading to activation of signaling pathways and modification of a variety of cellular functions [2]. Intracellularly, GPR4 couples to several Gα protein subunits [3], triggering the activation of their respective downstream signaling pathways, α_s_/cAMP [1], α_q/11_/IP/Ca^2+^ [4], α_12/13_/Rho/ROCK [5]. Recent studies have shown a high correlation between the transcriptional regulation of GPR4 and endothelial marker genes and highlighted a role for endothelial cells (ECs) in sensing local tissue acidosis via GPR4 [6, 7, 8]. GPR4 reaches its maximum activation at about pH 6.8 and is silent at pH above 7.8 [1]. GPR4 is mainly expressed in vascular ECs [6, 9], although it can be found in pericytes surrounding the vasculature [10, 11] and in fibroblasts [12]. The latter can transdifferentiate from endothelial cells through inflammation‐driven endothelial‐to‐mesenchymal transition (EndoMT) [13].

IBD consists of two main phenotypes: ulcerative colitis (UC) and Crohn's disease (CD). In 2017, there were 6.8 million cases of IBD worldwide [14]. IBD is characterized by severe mucosal tissue damage. The pathogenesis of IBD is linked to genetic susceptibility [15], intestinal microbiota [16], environmental factors [17], and immunological abnormalities [18]. Acidification in the intestinal lumen is implicated in the pathogenesis and progression of IBD [19, 20]. Fecal fluid from patients with severe UC is characterized by low fecal pH, the presence of bicarbonate, and lactate [21]. Low tissue pH occurs in inflammation‐induced tumors due to hypoxia, low perfusion, and the presence of glycolytic metabolites [22].

Gene expression databases show moderate expression of GPR4 in small intestine and colon. Intestinal tissue from IBD patients shows increased expression of GPR4, especially in inflamed tissue, compared to intestinal tissue from healthy individuals [23], reflecting inflammation linked to an angiogenic response. In addition, the fibrotic area in the intestinal wall of IBD patients shows increased expression of GPR4 compared to the non‐fibrotic resection margin [12]. A positive correlation between GPR4 and the expression of markers of fibrosis (e.g., alpha smooth muscle actin and several procollagens) has been demonstrated [12]. In mice, *Gpr4* deficiency protects against inflammation [23] and fibrosis in the spontaneous interleukin 10 knockout (*Il10* ^‐/‐^) colitis model [12]. The beneficial effect of *Gpr4* deficiency has also been demonstrated in the chronic DSS‐induced model [23].

Colorectal cancer (CRC) is one of the most frequently diagnosed cancers. Chronic intestinal inflammation is a risk factor that promotes the development of CRC. The risk of colitis‐associated cancer in IBD is increased after a long duration of illness and chronic active disease [24, 25]. In tumors, protons (H^+^) are accumulated by increased anaerobic and aerobic glycolysis (Warburg effect). Lactic acid is released, leading to an acidic tumor microenvironment (TME) [26, 27], where a local pH below 7.0 is common. This contributes to malignant progression, tumor growth, metastasis, metabolic rewiring, and decreased immune surveillance [28, 29, 30, 31].

Enhanced expression of *GPR4* mRNA is detected in human cancers, including gastric cancer and colon adenocarcinoma [32], and CRC [33]. In patients with CRC, GPR4 is increased in tumor tissue, and high GPR4 expression correlates with late‐stage tumors and poor overall survival [33].

Loss of GPR4 in mice is linked to reduced tumor angiogenesis and tumor development [6]. Histology of murine tumors revealed changes in vessel morphology, vessel length, and density. Decreased angiogenesis is associated with a decreased endothelial cell response to vascular endothelial growth factor, key to vascular response to hypoxia [6]. Similarly, it was recently shown that intestinal inflammation, colon cancer development, and tumor angiogenesis were decreased in *Gpr4*‐deficient animals compared to wild‐type (WT) controls [34].

While GPR4 is predominantly expressed in endothelial cells, previous studies have also highlighted a possible direct role for GPR4 in regulating cancer cell behavior. For example, ectopic overexpression of GPR4 altered the motility of melanoma cells, inhibited cell migration in B16F10 [35] and increased it in SK‐Mel‐28 [36], but did not induce a more invasive phenotype. In NIH3T3 cells, GPR4 expression induced a malignant phenotype [37]. Knockdown of GPR4 in HCT116 and HT29 CRC cell lines led to reduced growth and migration [33]. Thus, GPR4 activity may contribute to oncogenic progression across a many cancers.

In this study, we aimed to better understand the physiological role of GPR4 in tumorigenesis in animal models of colon cancer. *Gpr4*‐deficient and WT mice were used in the subcutaneously (*s.c*.) MC38 injection model and the AOM/DSS‐induced colon cancer model. Our data show that the absence of functional GPR4 in the tumor microenvironment reduces colon cancer growth. Moreover, the smaller tumors in *Gpr4*‐deficient mice were associated with higher numbers of NK cells but lower numbers of neutrophils and macrophages.

## 2 Materials and methods

### 2.1 Animals

All animal experiments were performed according to the ARRIVE criteria. *Gpr4* ^*−/−*^ mice (B6.Balb/c‐GPR4tm1Lud and C57BL/6 background) were provided by Novartis, Basel. The animal experiment protocol was approved by the Veterinary Authority of the canton of Zurich (registration number ZH211/2020 and ZH113/2021). Mice with an identical genetic background (littermates) were used in all experiments.

For tumor induction with MC38 (RRID:CVCL_B288, a murine colon adenocarcinoma cell line), a total of 29 WT mice were compared with 16 *Gpr4* ^*−/−*^ mice. Female mice aged 10–13 weeks and weighing approximately 20 g were used for the experiment. For tumor induction with AOM/DSS, a total of 24 female WT mice were compared with 7 female *Gpr4* ^*−/−*^ mice, aged 10–13 weeks and weighing approximately 20 g were used for the experiment.

The animals were co‐housed wherever possible, and bedding was exchanged among the cages to minimize potential effects of microbiota variation. All the animals were housed in a specific pathogen‐free facility as previously described in [38]. The animals were kept in type II long clear transparent individually ventilated cages (IVCs, 365 mm × 207 mm × 140 mm, Allentown, New Jersey, USA) with autoclaved dust‐free bedding and tissue papers as nesting material. They were fed a pelleted and extruded mouse diet (R/M–H Extrudat, ssniff Spezialdiäten, Soest, Germany) *ad libitum*. The light/dark cycle in the room was provided through natural daylight (sunrise: 07:00 h, sunset: 18:00 h). The mice were weighed at 10:00 h every morning. The temperature was set to 21 ± 1 °C, with a relative humidity of 55 ± 5% and 75 complete changes of filtered air per hour (filter: Megalam MD H14, Camfil, Zug, Switzerland).

### 2.2 Tumor models

MC38 cells were originally obtained from Dr. J. Schlom (NIH) in 1997; cells were expanded into low passage number and working stocks frozen down [39]. For injection models, MC38, MC38‐green fluorescent protein (GFP), and MC38‐luciferase (all three cell lines donated by Lubor Borsig, Institute of Physiology, University of Zurich, Zurich, Switzerland) were used. Cells were expanded to low passages, and working stocks were frozen. All cell lines were tested using the EZ‐PCR Mycoplasma Kit (20‐700‐20; Lucerna Chem AG, Switzerland) and found to be free of mycoplasma, while no further cell authentication assays were performed. Cells were passaged two to three times after thawing prior to implantation into mice.

Cells were suspended in culture medium, mixed 1 : 2 with Matrigel and 300 000 cells, and injected *s.c*. into the flank of mice. In the *s.c*. model, tumor growth was measured every 2 days using a digital caliper. Tumor volume was calculated using the ellipsoid formula: 4/3 × 3.14 × length/2 × (width/2)^2^, where the shorter dimension was used as the width and depth. The duration of the experiment was coordinated according to the termination criteria established by the veterinary office: All mice in an experimental group were euthanized before the tumors of one or more animals in the experimental group reached a volume of 1 cm^3^ or a length of 2 cm. Mice with MC38 and MC38‐luciferase were euthanized 14 days after injection. MC38‐GFP mice were euthanized 16 days after injection. A total of 11 WT mice were compared with 8 *Gpr4* ^−/−^ mice using MC38‐luciferase cells. A total of 6 WT mice were compared with 5 *Gpr4* ^−/−^ mice using MC38‐GFP cells. A total of 12 WT mice were compared with 3 *Gpr4* ^−/−^ mice using MC38 cells.

The AOM/DSS model of colitis‐induced cancer was performed according to a modified protocol by Neufert *et al*. [40]. Briefly, colitis was induced with DSS (36–50 kDa; MP Biomedicals, Santa Ana, CA, USA) as previously described in [12]. For chronic colitis, WT and *Gpr4* ^−/−^ mice were administered 4 cycles of 3% DSS in drinking water *ad libitum* for 7 days, followed by 10 days of normal drinking water. AOM (Sigma Aldrich; St. Louis, MO, USA, 10 mg/kg in saline) was injected intraperitoneally (*i.p*.) on day 1 and day 9 of each DSS cycle. Mouse body weight and clinical phenotype were assessed daily. Before the third DSS cycle and at the end of the experiment, mice were anesthetized, and a colonoscopy was performed to monitor tumor development and inflammation. After the last DSS cycle, all animals were allowed to recover for 3 weeks and then sacrificed for sampling.

### 2.3 Assessment of colonoscopy and histological score in mice

Prior to endoscopic assessment, the animals were anesthetized *i.p*. with a mixture of 90–120 mg ketamine (Narketan 10%, Vétoquinol AG, Bern, Switzerland) and 8 mg xylazine (Rompun 2%; Bayer; Leverkusen, Germany) per kg body weight, and examined with the Tele Pack Pal 20 043 020 (Karl Storz Endoskope, Tuttlingen, Germany) as previously described in [41].

For histological scoring, colon and small intestine were rolled into Swiss rolls, fixed in paraformaldehyde solution (4% in PBS; Santa Cruz Biotechnology, Dallas, TX, USA), embedded, stained with hematoxylin and eosin (HE), and scored as described [42]. Briefly, mice were scored individually. Histology was performed by an independent investigator blinded to the type of treatment. Histology was scored as follows as previously described in [38]:

Epithelium (E) 0: normal morphology; 1: loss of goblet cells; 2: loss of goblet cells in large areas; 3: loss of crypts; 4: loss of crypts in large areas.
Infiltration (I) 0: no infiltrate; 1: infiltrate around the crypt base; 2: infiltrate extending to the *L. muscularis mucosae*; 3: extensive infiltrate extending to the *L. muscularis mucosae* and mucosal thickening with abundant edema; 4: infiltration of the *L. submucosa*.

The total histologic score is the sum of the epithelial and infiltration scores and ranges from 0 to 8 (total score = E + I).

### 2.4 Quantification of AP sites

DNA damage was determined using a colorimetric assay kit (ab211154; Abcam, Cambridge, UK) according to the manufacturer's instructions. Briefly, snap‐frozen whole colon resections from the AOM/DSS CRC model were disrupted in 400 μL lysis buffer in M tubes (Miltenyi Biotec, Bergisch Gladbach, Germany) using a gentleMACS tissue homogenizer (Miltenyi Biotec). The concentration of genomic DNA was determined by absorbance at 260 nm and 280 nm using a NanoDrop (Thermo Fisher Scientific, Waltham, MA, USA). 100 μg/mL of purified genomic DNA was diluted in TE buffer. 5 μL was combined with 5 μL aldehyde reactive probe solution. DNA was precipitated with glycogen, sodium acetate solution, and absolute EtOH at −20 °C for 30 min. After centrifugation, the DNA pellet was washed three times with 70% ethanol, air dried, and resuspended in TE (1 μg/mL). DNA binding solution, streptavidin‐enzyme conjugate, and substrate solution were added to a 96‐well plate and incubated at room temperature for ≤20 min on an orbital shaker. Stop solution was added and read on a microplate reader at OD 450 nm. The OD reading, corrected for the absorbance value of the blank, was applied to the standard curve for the number of AP sites/10^5^ bp.

### 2.5 Tumor cell isolation

Tumor was minced into small pieces and digested in 6 mL HBSS prepared from the solid salts (Sigma Aldrich, Hanks' Balanced Salts, H2387‐10X1L). 0.5 mg·mL^−1^ collagenase type IV (Gibco, 17 104‐019) and 0.05 mg·mL DNase I (Roche; Basel, Switzerland, 10 104 159 001) were added for 15 min at 37 °C with shaking. After incubation, the samples were sheared using a syringe and 18 G needle and then passed through a 70 μm cell strainer. The digestion was stopped by adding 3 mL fetal calf serum (FCS) low Ig (PAN Biotech; Aidenbach, Germany, P30‐2802) and 10 mL PBS. The supernatant was removed after centrifugation at 470 *g* for 5 min at 4 °C.

### 2.6 RNA isolation, complementary DNA (cDNA) synthesis, and qPCR

Total RNA was isolated from colon and ileum using the Maxwell RSC simplyRNA tissue kit (Promega, Madison, WI, USA, AS1340) as previously described in [41]. For all samples, lysis buffer from the kit was added to snap‐frozen resections, and samples were shredded in M tubes (Miltenyi Biotec) using a gentleMACS tissue homogenizer (Miltenyi Biotec). RNA concentration was determined by absorbance at 260 nm and 280 nm with a NanoDrop (Thermo Fisher Scientific). cDNA synthesis was performed using a High‐Capacity cDNA Reverse Transcription Kit (Applied Biosystems, Foster City, CA, USA) following the manufacturer's instructions. qPCR was performed using the TaqMan Fast Universal Master Mix (Applied Biosystems) on a QuantStudio™ 6 Flex Real‐Time PCR System, and results were analyzed with the SDS software (Applied Biosystems). For each sample, triplicates were measured, and glyceraldehyde‐3‐phosphate dehydrogenase (*Gapdh*) was used as an endogenous control. Results were analyzed using the ∆∆CT method. The following gene expression assays for mouse were used (Thermo Fischer Scientific): *Bcl2* Mm00477631_m1, *Fasl* Mm00438864_m1, *Il‐6* Mm00446190_m1, *Klrb1c* Mm07307455_s1, *Mmp9* Mm00442991_m1, and *Gapdh* 4352339E.

### 2.7 Flow cytometry

For single‐cell flow cytometry analysis, single cells from tumors were isolated. Surface antigens were stained with a mix of antibodies including a viability marker (Table S1) and incubated at 4 °C for 20 min as previously described in [38]. After washing with phosphate‐buffered solution (PBS) and centrifugation, all the samples were fixed. For fixation, BD Cytofix/Cytoperm (554722) and BD Perm/Wash (554723) were used following the manufacturer's instructions. The pellet was then resuspended in PBS. Intracellular antigens were stained with a mix of antibodies (Table S1). Data were acquired on a FACS LSR II Fortessa 4 L (BD) and analyzed with the FlowJo software (version 10.2).

### 2.8 Compensation controls

Two different bead kits were used for compensation controls as previously described in [38]: ArC Amine reactive compensation bead kit (Thermo Fisher Scientific, A10346) for the cell viability marker. For the remaining antibodies, the BD CompBeads kit anti‐Rat and anti‐Hamster (Becton Dickinson Pharmingen Biosciences, Franklin Lakes, NJ, USA, 552 845), and the BD CompBeads kit anti‐Mouse (Becton Dickinson Pharmingen Biosciences, 552 843) were used. All the compensation controls were prepared following the manufacturer's instructions.

### 2.9 Immunohistochemistry (IHC) and immunofluorescence

Specimens were fixed in 4% PBS‐buffered formalin, embedded in paraffin, and sectioned (3 μm) as previously described in [38]. H&E staining was performed using standard procedures. Tissues for IHC were deparaffinized, and antigen retrieval was performed in citrate buffer, pH 6.0 (Dako), at 98 °C for 30 min. Inhibition of endogenous peroxidases was performed by incubating tissue slides in 0.9% hydrogen peroxide for 15 min at RT. Horse serum (2.5%), to block non‐specific binding, was used for Ki67 staining for 1 h at RT, and mouse serum was used for NKp46 staining, a marker of NK cells. Primary antibodies were diluted in blocking solutions, and slides were incubated overnight at 4 °C. For IHC samples, a secondary HRP‐conjugated antibody (Vector Laboratories; Newark, CA, USA, ImmPRESS) was applied for 1 h at RT, and staining was visualized using the 3,3′‐diaminobenzidine (DAB) ImmPACT Peroxidase Substrate Brown Kit (Vector Laboratories). IHC specimens were counterstained with hematoxylin, dehydrated, and mounted. Ki67 was stained with a monoclonal rabbit anti‐mouse *Ki67/MKI67* (NB600‐1252; Novus Biologicals; Centennial, CO, USA, 1:100). For sections from the MC38 model, NK1.1 expressed by both NK cells and a fraction of NKT cells was stained with a rabbit anti‐mouse NK1.1/CD161 (clone E6Y9G, 39 197; Cell Signaling; Danvers, MA, USA, 1:200) antibody. For sections from the AOM/DSS model, NK‐cell protein expressed by both NK cells and NKT cells was stained with a monoclonal mouse anti‐mouse NKp46/NCR1 (NBP2‐11820, Novus Biologicals, 1:200) antibody. IHC for CXCL12/SDF‐1 was performed on a Ventana stainer (Roche) with a monoclonal mouse anti‐mouse (MAB350, R&D Systems; Minneapolis, MN, USA, 1:100), CK20 with a monoclonal rabbit anti‐mouse (ab64909, Abcam; Cambridge, UK, 1:100), and specimens were stained with hematoxylin using standard histological techniques. Staining was examined using the Imager Z2 microscope and the software ZEN. 20× magnification pictures from at least three representative areas from each section were taken using transmission light microscopy. The relative quantity of the according antigen was analyzed using the fiji software (1.52a, NIH). The total tissue area was determined by converting the image into an 8‐bit image type and adjusting the B&W threshold. The area covered with DAB was determined by setting thresholds to select the brown color (for KI67: Hue 125–190, Brightness 190–255). The quantity of the according antigen was calculated as area of brown color/total tissue area.

### 2.10 Statistical analysis

Statistical analysis was performed as indicated in the figure legends using GraphPad Prism (v 9.4.1) as previously described in [38]. Differences were considered significant at a *P*‐value <0.05, as indicated in the figures. Results are presented as mean ± standard deviation (SD) or ± standard error of the mean (SEM), as indicated in the figure legends. Differences were considered significant at a *P*‐value of <0.05 (*), highly significant at a *P*‐value of <0.01 (**) and very highly significant at a *P*‐value of <0.001 (***).

## 3 Results

### 3.1 Loss of GPR4 reduces tumor burden in the MC38 *s.c.* injection model

**Fig. 1:**
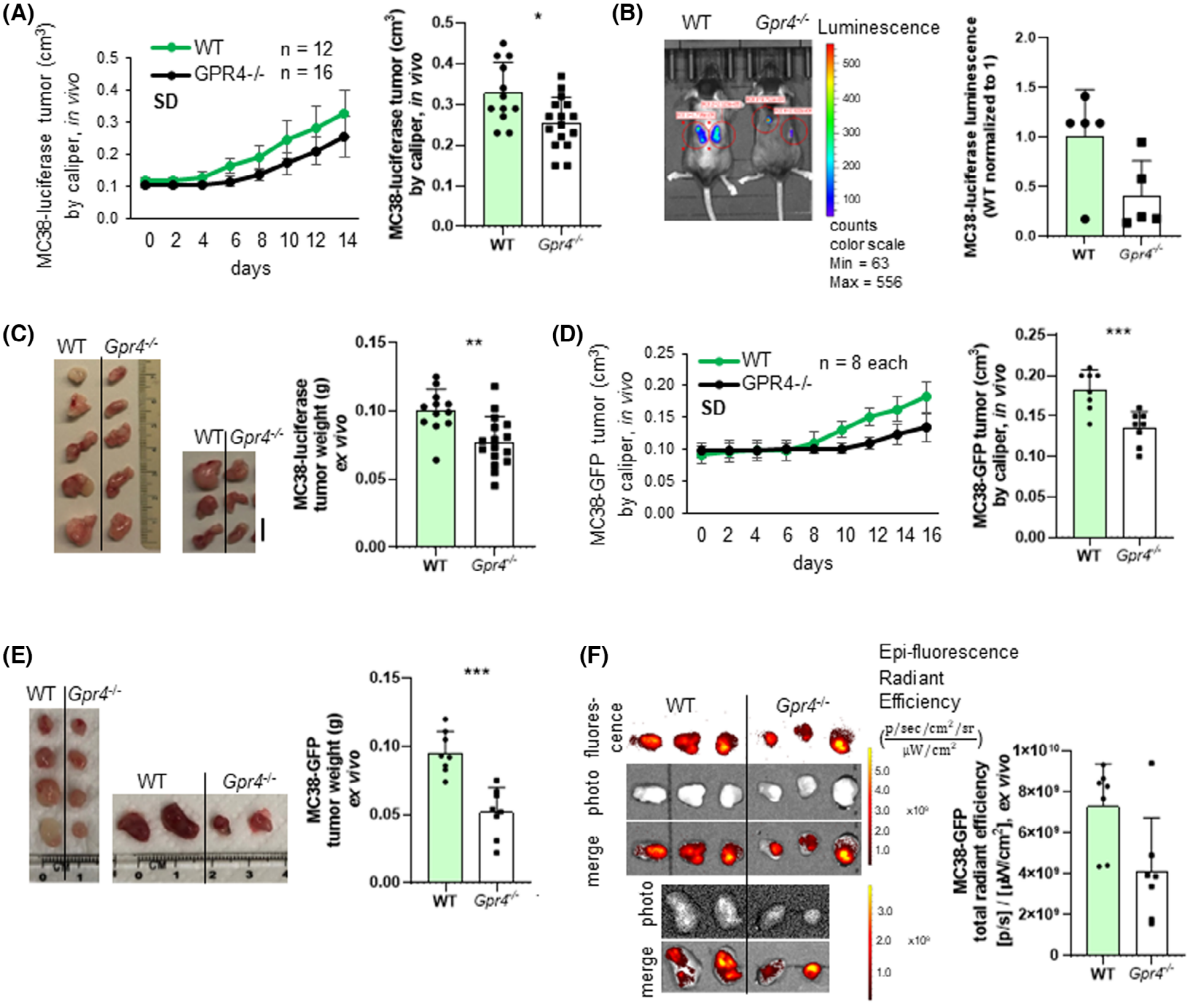
Decreased tumor size in *Gpr4* ^−/−^ compared with WT mice. (A–D) 300 000 MC38 tumor cells expressing luciferase were injected *s.c*. into WT and *Gpr4* ^−/−^, mice were euthanized 14 days after injection. (E–H) 300 000 MC38 tumor cells expressing GFP were injected and mice were euthanized after 16 days. (A) MC38_luciferase tumor volume over time (*P* = **, *n* = 5 each). (B) *In vivo* total radiant efficiency of tumor development on the last day of the experiment (*P* = 0.0873, *n* = 12 for WT and 16 for *Gpr4* ^−/−^). (C) Tumor size and weight. Scale bar 10 mm (*P* = **, *n* = 12 for WT and 16 for *Gpr4* ^−/−^). (D) MC38_GFP tumor volume over time (*P* = ***, *n* = 8 each). (E) Tumor size and weight (*P* = ***, *n* = 8 each). (F) *Ex vivo* total radiant efficiency (*P* = 0.530, *n* = 7 each). (A, C, E) Normal distribution (Shapiro–Wilk test), unpaired *t*‐test. (B, F) Non‐parametric distribution (Shapiro–Wilk test), Mann–Whitney test. Error bars indicate SD. WT, wild‐type, s.c., subcutaneous.

To study the role of GPR4 in colon cancer progression, we injected MC38 colon adenocarcinoma cells into mice lacking GPR4 expression. We used MC38‐luciferase cells to monitor tumor burden in *Gpr4* ^−/−^ mice vs. WT (Fig. S1A). We observed slower tumor growth and significantly reduced tumor size *in vivo* in *Gpr4* ^−/−^ mice compared to WT mice (Fig. 1A). Analysis of luciferase activity showed a trend towards decreased luminescence in *Gpr4* ^−/−^ mice (Fig. 1B). Tumor weight was determined *ex vivo* and shown to be significantly reduced in *Gpr4* ^−/−^ mice compared to WT mice (Fig. 1C). To further validate these observations, we repeated these experiments using a different MC38 line, which carries a GFP reporter instead of luciferase (MC38‐GFP) (Fig. S1B). Results from these analyses confirmed slower tumor growth, significantly reduced tumor size *in vivo* (Fig. 1D), and tumor weight *ex vivo* (Fig. 1E) in *Gpr4* ^−/−^ mice. Evaluation of green fluorescence showed a trend towards decreased emission in *Gpr4* ^−/−^ mice (Fig. 1F).

### 3.2 Loss of GPR4 regulates immune infiltration within the tumor

**Fig. 2:**
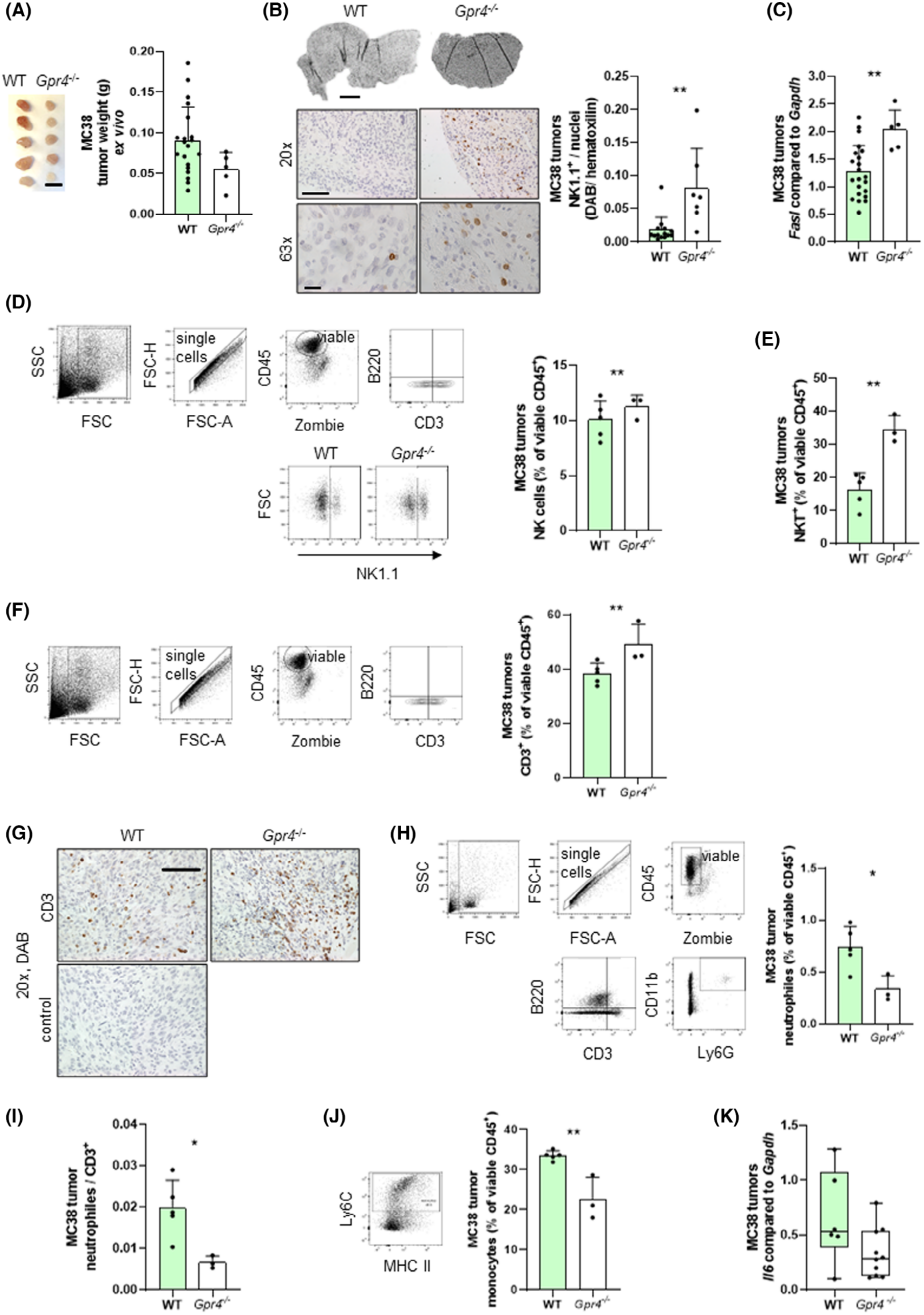
Increased number of NK1.1^+^ cells in *Gpr4* ^−/−^ compared to WT mice. 300 000 MC38 tumor cells were injected *s.c*. into WT and *Gpr4* ^−/−^, mice were euthanized 14 days after injection. (A) Tumor size and weight. Scale bar 10 mm (*P* = 0.0866, *n* = 20 for WT and 5 for *Gpr4* ^−/−^). (B) IHC and quantification for NK1.1^+^ cells (DAB brown, hematoxylin blue). Scale bar for panorama 500 μm, for 20× 100 μm, and for 63× 20 μm (*P* = **, *n* = 14 for WT and 7 for *Gpr4* ^−/−^). (C) qPCR, *Fasl* (*P* = **, *n* = 22 for WT and 5 for *Gpr4* ^−/−^). (D–F) Flow cytometry and quantification. (D) CD3^−^ NK cells (*P* = 0.5714, *n* = 5 and 3). (E) CD3^+^ NKT cells (*P* = **, *n* = 5 for WT and 3 for *Gpr4* ^−/−^). (F) CD3^+^ cells (*P* = *, *n* = 5 for WT and 3 for *Gpr4* ^−/−^). (G) IHC and quantification for CD3^+^ cells (DAB brown, hematoxylin blue, *P* = ***, *n* = 4 each). Scale bar 100 μm. (H–J) Flow cytometry and quantification. (H) Neutrophiles (*P* = *, *n* = 5 for WT and 3 for *Gpr4* ^−/−^). (I) Neutrophiles/CD3^+^ (*P* = *, *n* = 5 for WT and 3 for *Gpr4* ^−/−^). (J) Monocytes (*P* = **, *n* = 5 for WT and 3 for *Gpr4* ^−/−^). (K) qPCR, *Il6* (*P* = 0.0651, *n* = 6 for WT and 10 for *Gpr4* ^−/−^). (A, C, E, F, H–K) Normal distribution (Shapiro–Wilk test), unpaired *t*‐test. (B, D) Non‐parametric distribution (Shapiro–Wilk test), Mann–Whitney test. Error bars indicate SD. DAB, 3,3′‐diaminobenzidine; IHC, immunohistochemistry; s.c., subcutaneous; WT, wild‐type.

Tumors in *Gpr4* ^−/−^ mice show reduced growth relative to WT, suggesting that cells within the microenvironment constrain MC38 cancer cell growth in this model. We therefore studied immune cell infiltration in tumors of *Gpr4* ^−/−^ and WT mice. These experiments were carried out using standard MC38 cells without luciferase and GFP (Fig. S1C). Decreased tumor weight in *Gpr4* ^−/−^ mice was confirmed at *postmortem* (Fig. 2A). IHC was used to assess the presence of NK1.1^+^ cells expressing the NK1.1^+^ cell marker in tumor samples. The number of NK1.1‐positive cells was significantly increased in *Gpr4* ^*−/−*^ mice compared to WT mice (Fig. 2B). Additionally, tumors in *Gpr4* ^−/−^ mice showed significantly increased *Fasl* mRNA expression (Fig. 2C), indicating enhanced cytotoxic cell activity, which is in line with the augmented presence of NK cells. Flow cytometry analysis of the tumor infiltrate confirmed an increase in both NK (Fig. 2D) and NKT (Fig. 2E) cells in the tumors of *Gpr4* ^*−/−*^ mice.

NK cells are innate lymphocytes that play a key role in cancer immunity, and strategies have been developed to boost their presence and function in tumor tissue [43, 44]. IL2 is a cytokine that supports NK cell proliferation and cytolytic activity, and various forms of IL2 are used in clinical trials in oncology [44]. Interestingly, endogenous IL2 appears to be inactivated in the acidic tumor microenvironment [45]. We therefore investigated IL2 expression and protein levels in MC38 tumors.

**Fig. 3:**
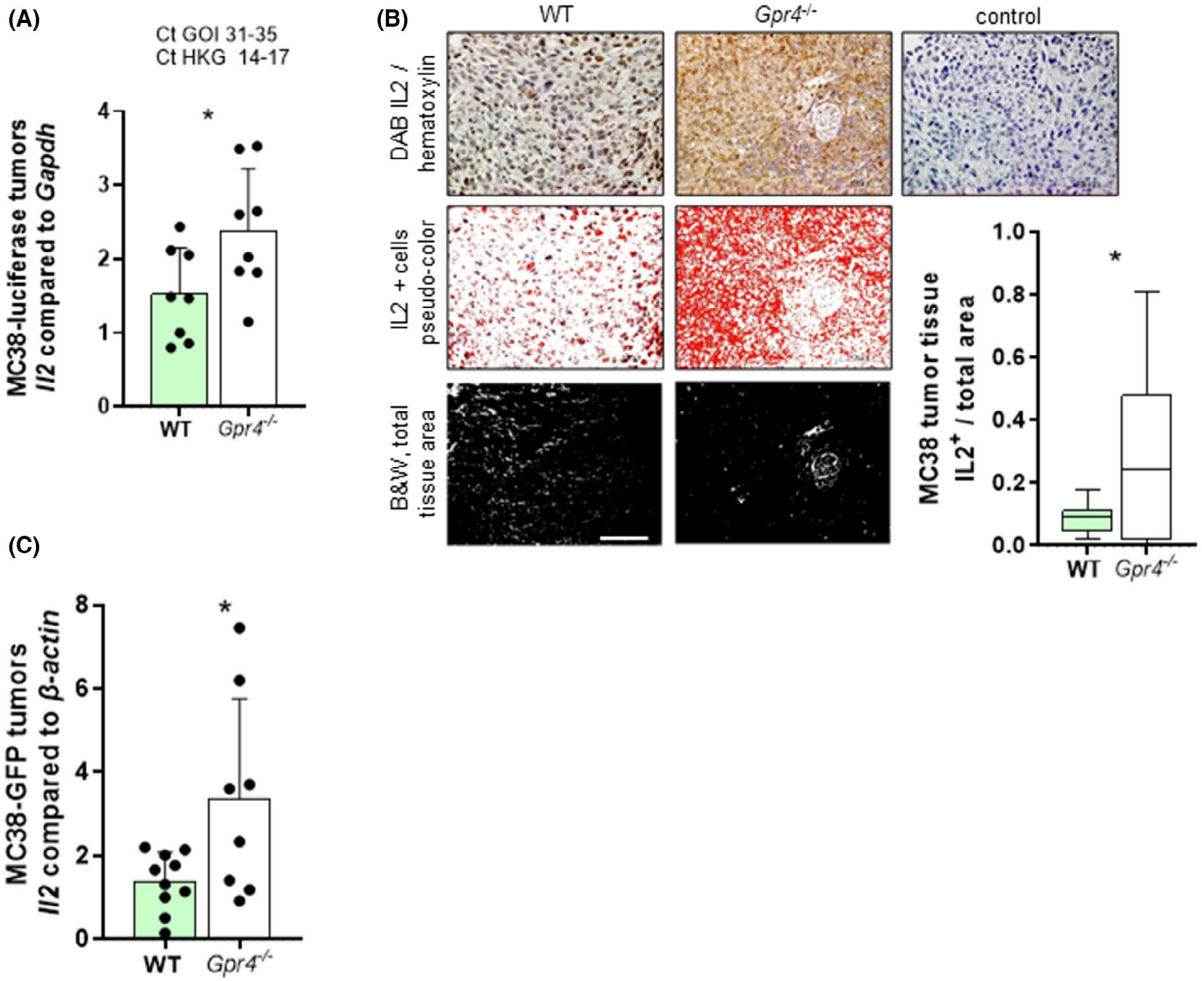
Increased *Il2* in *Gpr4* ^−/−^ compared with WT mice. (A and B) 300 000 MC38 tumor cells expressing luciferase were injected *s.c*. into WT and *Gpr4* ^−/−^, mice were euthanized 14 days after injection. (C) 300 000 MC38 tumor cells expressing GFP were injected and mice were euthanized after 16 days. (A) qPCR, *Il2* (*P* = *, *n* = 8 each). (B) IHC and quantification for IL‐2 (DAB brown, hematoxylin blue). Scale bar 100 μm (*P* = *, *n* = 15 for WT and 8 for *Gpr4* ^−/−^). (C) qPCR, *Il2* (*P* = *, *n* = 10 for WT and 8 for *Gpr4* ^−/−^). (A–C) Normal distribution (Shapiro–Wilk test), unpaired *t*‐test. Error bars indicate SD. DAB, 3,3′‐diaminobenzidine; IHC, immunohistochemistry; s.c., subcutaneous; WT, wild‐type.

Indeed, MC38‐luciferase containing tumors in *Gpr4* ^−/−^ mice showed significantly increased *Il2* mRNA expression compared to WT (Fig. 3A). IHC revealed significantly enhanced staining for IL2 protein in MC38‐luciferase tumor tissue from *Gpr4* ^*−/−*^ mice compared to WT mice (Fig. 3B). Confirmatively, tumors containing MC38‐GFP cells in *Gpr4* ^−/−^ mice showed significantly increased *Il2* mRNA expression (Fig. 3C).

With respect to other cells of the immune infiltrate, CD3‐positive T cells overall were significantly increased in tumor tissue of *Gpr4* ^*−/−*^ mice compared to WT (Fig. 2F, single, viable, CD45^+^, B220^−^, CD3^+^, panel 1 Table S1 and Fig. 2G). In contrast to the infiltration of NK and T cells, MC38 tumors showed a significantly reduced infiltration of neutrophils (Fig. 2H, single, viable, CD45^+^, B220^−^, CD3^−^, CD11b^+^, Ly6G^+^, panel 2 Table S1). The increased number of CD3^+^ lymphocytes concomitant with reduced neutrophils in tumor tissue of *Gpr4* ^−/−^ animals leads to a significantly reduced neutrophil/CD3^+^ cell ratio compared to WT (Fig. 2I). MC38 tumors also showed a significantly reduced infiltration of monocytes (Fig. 2J, single, viable, CD45^+^, B220^−^, CD3^−^, Ly6G^−^, MHC II^+^, Ly6C^+^, panel 2 Table S1). Consistent with the reduced number of macrophages, MC38 tumors injected into *Gpr4* ^−/−^ mice showed significantly reduced *Il6* expression (Fig. 2K).

Taken together, results from the heterotopic MC38 model showed that the absence of GPR4 in host animals significantly attenuated tumor progression. This effect was paralleled by an increased presence of NK and NKT cells, indicating enhanced elimination of tumor cells, and a decreased presence of macrophages and neutrophils, demonstrating reduced inflammation in *Gpr4* ^−/−^ mice. The increase of NK and NKT cells observed may be linked to locally augmented IL2 levels in tumor tissue.

### 3.3 Loss of GPR4 reduces tumor burden in the AOM/DSS model

The MC38 *s.c*. injection model allows us to assess engraftment and growth of established colon cancer cells, yet it does not allow us to study the role of GPR4 in early tumor development, which in the case of CRC can be driven by inflammation. To overcome these limitations, we used the AOM/DSS model of colitis‐induced cancer, combining the administration of a mutagenic agent with an inflammatory agent. As described in Material and Methods, repeated *i.p*. administration of the mutagenic agent AOM, which exerts colonotropic carcinogenicity, and the inflammatory agent DSS in drinking water causes spontaneous tumor formation and rapid growth of multiple colon tumors in mice within weeks. To chronify colitis, mice were administered 4 cycles of DSS. Earlier studies suggest that GPR4 supports inflammation; we therefore hypothesized that inflammation and spontaneous tumor development would be reduced in the AOM/DSS model of colitis‐induced cancer in *Gpr4*‐deficient animals. In the novel work presented here, a total of 24 WT mice were compared with 7 *Gpr4* ^*−/−*^ mice in this model (Fig. S1D). In line with our hypothesis, *Gpr4* ^*−/−*^ mice tolerated AOM/DSS treatment significantly better than WT mice, indicated by significantly increased weight gain, a decreased clinical disease activity score, significantly decreased spleen weight, significantly increased colon length, and a significantly ameliorated histologic score in both colon and small bowel, reflecting protection from inflammatory processes (Fig. S2A–F).

Progress coloscopy before the third DSS cycle revealed a clearly visible change in the colonic mucosa that indicated increased dysplasia in WT mice compared to *Gpr4* ^−/−^ mice (Fig. S3A). Histologic sections of tissue with dysplasia showed a clear alteration of the *lamina propria* in H&E staining, indicating a loss of crypt symmetry. After completion of the four DSS cycles, significantly increased *Bcl2* mRNA expression was detected in whole colon tissue from WT mice compared to *Gpr4* ^−/−^ mice (Fig. S3B). IHC was used to assess the presence of the proliferation marker Ki67 and the marker CXCL12 in whole colon tissue. Ki67 signals could be detected at the crypt base regardless of whether the tissue was normal or abnormal, indicating the presence of stem cells. The number of Ki67‐positive cells was significantly increased in tumor areas compared to normal tissue (Fig. S3C). A CXCL12 expression gradient with a maximum crypt top was confirmed. CXCL12 sensitizes cells to anoikis and minimizes tumor progression in normal tissue [46]. Areas of reduced CXCL12 expression were associated with dysplasia or tumors (Fig. S3D).

**Fig. 4:**
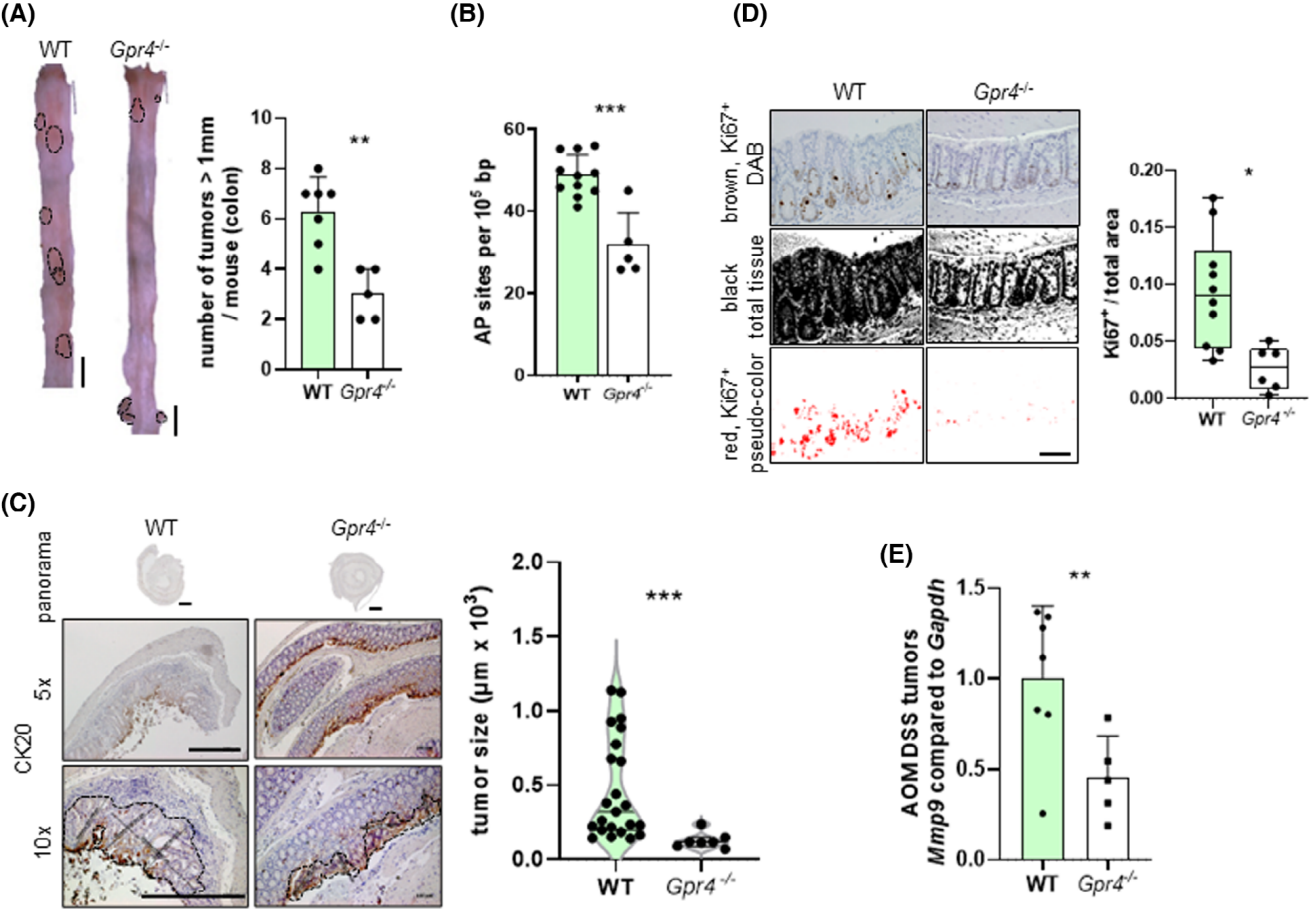
Decreased number of tumors and AP sites in *Gpr4* ^−/−^ compared with WT mice upon AOM DSS colitis. (A) Tumor size and number of tumors, colon. Scale bars 5 mm (*P* = **, *n* = 7 for WT and 5 for *Gpr4* ^−/−^). (B) AP sites, colon (*P* = ***, *n* = 11 for WT and 5 for *Gpr4* ^−/−^). (C) IHC and quantification, CK20, tumor size. Scale bar for panorama 1 mm, for 5× and 10× 500 μm each (*P* = ***, *n* = 23 for WT and 7 for *Gpr4* ^−/−^). (D) IHC and quantification, Ki67. Scale bar 100 μm (*P* = **, *n* = 10 for WT and 6 for *Gpr4* ^−/−^). (E) qPCR, *Mmp9* (*P* = *, *n* = 7 for WT and 5 for *Gpr4* ^−/−^). (A, B, D, E) Normal distribution (Shapiro–Wilk test), unpaired *t*‐test. (C) Non‐parametric distribution (Shapiro–Wilk test), Mann–Whitney test. Error bars indicate SD. AP, apurinic/ap yrimidinic; AOM, azoxymethane; DAB, 3,3′‐diaminobenzidine; DSS, dextran sodium sulphate; IHC, immunohistochemistry; WT, wild‐type.

Next, we evaluated the tumor burden in WT mice compared to *Gpr4* ^−/−^ mice in the AOM/DSS model. We observed a significantly reduced number of tumors (Fig. 4A) and a significantly reduced number of AP sites in *Gpr4* ^−/−^ mice compared to WT mice (Fig. 4B; Fig. S4). Evaluation of tumor size revealed significantly smaller tumors (Fig. 4C) and a reduced number of Ki67‐positive cells (Fig. 4D) in *Gpr4*‐deficient mice compared to WT mice. Previous studies have shown that a GPR4 antagonist decreased *Mmp9* mRNA expression [47]. In the current study, we hypothesized that MMP9, a potential biomarker for several cancers and a protein with an important role in cancer progression, would be reduced in the AOM/DSS model of colitis‐induced cancer in *Gpr4*‐deficient animals. Tissue samples in *Gpr4* ^−/−^ mice showed significantly decreased *Mmp9* mRNA expression (Fig. 4E).

**Fig. 5:**
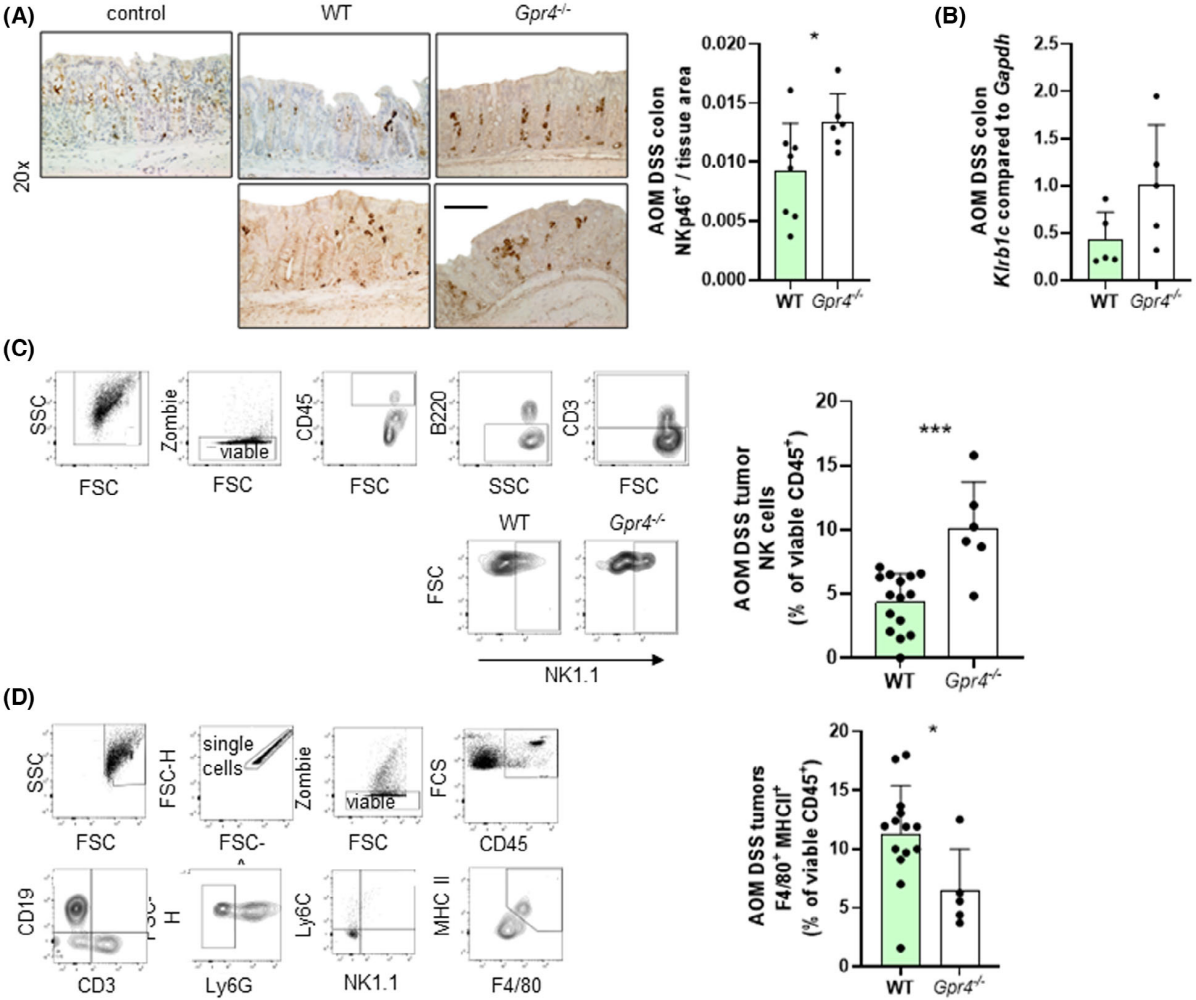
Increased number of NK cells in *Gpr4* ^−/−^ compared to WT mice upon AOM DSS colitis. (A, C) Whole colon. (B, D) Tumor tissue. (A) IHC and quantification, NKp46^+^ cells. Scale bar 100 μm (*P* = *, *n* = 8 for WT and 6 for *Gpr4* ^−/−^). (B) qPCR, *Klrb1c* (*P* = 0.0955, *n* = 5 each). (C) Flow cytometry and quantification, tumor, NK cells (*P* = ***, *n* = 15 for WT and 6 for *Gpr4* ^−/−^). (D) Flow cytometry and quantification, tumor, macrophages (*P* = *, *n* = 5 each). (A–D) Normal distribution (Shapiro–Wilk test), unpaired *t*‐test Error bars indicate SD. WT, wild‐type, IHC, immunohistochemistry, AOM, azoxymethane, DSS, dextran sodium sulphate.

IHC was used to assess the presence of NK cell protein NKp46^+^ cells in whole colon tissue. As observed in the *s.c*. injection model, the number of NKp46‐positive cells within tumors was significantly increased in *Gpr4* ^*−/−*^ mice compared to WT (Fig. 5A). Confirmatory, tumors in *Gpr4* ^−/−^ mice showed significantly increased killer cell lectin‐like receptor subfamily B, member 1 (*Klrb1c*) mRNA expression (Fig. 5B). In addition, flow cytometry confirmed an increase in NK cells in the tissue samples of *Gpr4* ^*−/−*^ mice (based on markers for viable, CD45^+^, B220^−^, CD3^−^, NK1.1^+^, panel 1 Table S1, Fig. 5C). As observed for the MC38 cell model, tumors showed a significantly reduced infiltration of macrophages (Fig. 5D, based on markers for single, viable, CD45^+^, B220^−^, CD3^−^, Ly6G^−^, Ly6C^−^, NK1.1^−^, MHCII^+^, F4/80^+^, panel 3 Table S1). Taken together, our results from the orthotopic AOM/DSS model of CRC showed that GPR4 deficiency significantly attenuated spontaneous tumor formation and tumor progression, supporting and extending the results from the MC38 model. In both models, the beneficial effects on tumor burden observed in *Gpr4* ^*−/−*^ mice correlate with an increased presence of NK cells and a reduction in the number of tumor‐associated macrophages and neutrophils.

## 4 Discussion

The results of this study show that loss of pH‐sensing receptor GPR4 reduces colorectal cancer cell growth *in vivo*, both in a model ectopically implanted MC38 colon carcinoma cells, as well as during spontaneous tumor development and growth in the AOM/DSS model. Our results align well with recent observations describing reduced angiogenesis and tumor growth in orthotopic cancer cell transplantation models with GPR4 deficient animals [6], and reduced tumor proliferation, tumor burden, and angiogenic microvessel density in an AOM/DSS model of colitis‐associated cancer [34].

GPR4 is predominantly expressed in ECs and to some degree in pericytes [6, 9, 10, 12, 32], and the available data underscore the important role that the local microvascular system plays in tumor growth [48]. While initial studies focused on the role of GPR4 in supporting tumor angiogenesis [6], more recent data suggest a broader role of the endothelium in sculpting the tumor microenvironment: Indeed, the blood vessel endothelium may allow lymphocyte extravasation, while endothelial cells act as immune modulators secreting lymphocyte‐attracting chemokines and inflammatory mediators [49]. In some cases, ECs may act as antigen‐presenting cells to support an adaptive immune response [50, 51].

GPR4 was shown to be required for the production of VEGF and for the angiogenic response to VEGF [6]. VEGF is a well characterized target for therapies based on “vascular normalization” which aim to make tumor tissue better accessible for treatment [48, 52]. We hypothesize that GPR4 inhibition, given its role in the VEGF response, supports vascular normalization. Further experiments assessing the status of the tumor vasculature in detail are expected to shed light on this question.

Our data suggest that the absence of GPR4 in the tumor‐bearing host may affect tumorigenesis through two parallel events: decreased inflammation and increased anti‐tumor responses. First, *Gpr4* deficiency protects against inflammation in several animal models of intestinal inflammation [12, 23]. Inflammation, especially chronic inflammation, plays a central role in tumorigenesis and metastasis [53]. Since inflammation, tissue destruction, and subsequent tissue regeneration are reduced in *Gpr4*‐deficient animals, it stands to reason that the rate of tumorigenesis is also reduced.

It is a remarkable finding of this study that tumors grown in *Gpr4* ^−/−^ animals exhibit increased tumor tissue infiltration by NK cells and CD3‐positive T cells compared to WT. Associated with this cell influx, we observed increased *Fasl* mRNA expression in tumor samples from *Gpr4* ^*−/−*^ mice. Fasl is expressed by cytotoxic T cells and NK cells and induces apoptosis [54, 55]. NK cells play an important role in the first line of defense against tumors [56] and strategies to augment NK cell presence and function in tumor tissue are tested in the clinical setting [43, 44]. Overall, our data indicate enhanced tumor cell clearance in *Gpr4* ^−/−^ mice compared to WT through increased cytotoxic cell activity. It is not yet fully established how the augmented presence of NK cells in the tumor infiltrate of *Gpr4* ^−/−^ animals is brought about. However, the observation that the NK cell supporting cytokine IL2 is significantly increased in tumor tissue of *Gpr4* ^−/−^ animals compared to WT may hold at least part of the response. Variants of IL2 are successfully used in the clinical setting to boost NK cell infiltration and anti‐tumor immunity [44, 57]. Of note, IL2 has reduced stability in the acidotic microenvironment of tumors [45]. Vascular normalization afforded through GPR4 inhibition may normalize tumor extracellular pH and stabilize IL2 protein. Diminished tumor acidosis may also directly enhance cytotoxic cell activity [54, 55, 58].

In parallel to the increased influx of NK cells and NKT cells in GPR4‐deficient animals, we observed a significant reduction of neutrophils and inflammatory monocytes in tumor tissue, accompanied by reduced pro‐inflammatory IL6 levels. Blockade of tumor‐associated macrophage function was suggested as an attractive strategy to improve NK cell‐based immunotherapies [59, 60], as macrophages were reported to activate NK cells under certain circumstances. Further, alternatively activated (M2) macrophages were reported to promote tumor progression and suppression of immune response [61].

The neutrophil response can both positively and negatively correlate with tumor development, and there is the emerging concept that particular cell states may be responsible for these divergent effects [62, 63]. Reduced neutrophil counts in the tumor infiltrate appear in line with enhanced cytotoxic cell activity [64]. In human tumors, a high neutrophil/lymphocyte ratio is associated with poor prognosis [65], and our data on the ratio of neutrophil/CD3^+^ cells indicate that this ratio is reduced in our murine models in *Gpr4* ^−/−^ animals compared to WT.

An important effector molecule released by tumor‐associated neutrophils, but also other cell types in the tumor microenvironment, is MMP9. This endopeptidase is responsible for the degradation of denatured collagens and basement membranes and is considered a biomarker of intestinal inflammation. It is also linked to tumor angiogenesis, invasion [66], and metastasis [67]. MMP9 inhibition is effective in an orthotopic xenograft model of CRC and has been proposed as a novel strategy for the treatment of metastatic CRC patients in its own right [68]. In line with the reduced neutrophil numbers in tumor samples, we observed reduced MMP9 expression in tumor tissue of *Gpr4* ^−/−^ animals compared to WT.

## 5 Conclusion

Our data demonstrate that the endothelial pH‐sensing receptor GPR4 regulates tumorigenesis, tumor growth, and modulates immune cell infiltration to tumor tissue. Consequently, the inhibition of GPR4 should be further investigated as a therapeutic option for CRC patients.

## Conflict of interest

LP, MB, PAR, EM, VB, FF, AG, TG, HE, CS, KH, GdeL, IA, CdeV, KS, and MH declare no competing interests. GR discloses consulting to Abbvie, Arena, Augurix, BMS, Boehringer, Calypso, Celgene, FALK, Ferring, Fisher, Genentech, Gilead, Janssen, Lilly, MSD, Novartis, Pfizer, Phadia, Roche, UCB, Takeda, Tillots, Vifor, Vital Solutions, and Zeller; speaker's honoraria from Abbvie, Astra Zeneca, BMS, Celgene, FALK, Janssen, MSD, Pfizer, Phadia, Takeda, Tillots, UCB, Vifor, and Zeller; educational grants and research grants from Abbvie, Ardeypharm, Augurix, Calypso, FALK, Flamentera, MSD, Novartis, Pfizer, Roche, Takeda, Tillots, UCB, and Zeller. Gerhard Rogler is cofounder and head of the scientific advisory board of PharmaBiome. All other authors have nothing to disclose.

## Author contributions

All authors approved the final submitted version of the manuscript. LP, MB: Data acquisition (IHC, qPCR, quantification of AP sites), data curation, and writing, original draft, review & editing. PAR: Data acquisition (scRNAseq), data curation, writing, review & editing. EM: Data acquisition (scRNAseq), data curation, writing, review & editing, final approval. VB: Data acquisition (qPCR), data curation, writing, review & editing. FF: Data acquisition (animal experiments, qPCR, scRNAseq), data curation, writing, review & editing. AG, TG, HE: Data acquisition (qPCR, *in vitro* cell culture experiments), data curation, writing, review & editing. CS: Data acquisition (IHC, qPCR), data curation, and writing, original draft. KH, GdeL, IA, CdeV: Data acquisition (scRNAseq), data curation, writing, review & editing. KS: Data acquisition (scRNAseq), data curation, writing, review & editing, final approval. MH: Data acquisition (animal experiments, IHC, qPCR, flow cytometry, quantification of AP sites, scRNAseq), data curation and writing, original draft, writing, review & editing, final approval. GR: Conceptualization, writing, review, and editing.

## Peer review

The peer review history for this article is available at https://www.webofscience.com/api/gateway/wos/peer‐review/10.1002/1878‐0261.70045.

## Supporting information


**Fig. S1.** Experimental setup. 300 000 MC38 tumor cells expressing (A) luciferase, (B) GFP, and (C) unmodified cells were injected *s.c*. into WT and *Gpr4* ^−/−^. (D) Tumor induction with AOM/DSS. AOM, azoxymethane, DSS, dextran sodium sulphate; s.c., subcutaneous; WT, wild‐type.

**Fig. S2.** Decreased inflammation in *Gpr4* ^−/−^ compared with WT mice upon AOM DSS colitis. (A) Body weight (*P* = **, *n* = 24 and 7). (B) Clinical score (*n* = 24 for WT and 7 for *Gpr4* ^−/−^). (C) Spleen weight (*n* = 1 each). (D) Colon length. Scale bar 10 mm (*P* = ***, *n* = 17 for WT and 7 for *Gpr4* ^−/−^). (E) Histological score, colon. Scale bar for 5 × 500 μm and for 20× 100 μm (*P* = *, *n* = 11 for WT and 6 for *Gpr4* ^−/−^). (F) Histological score, small bowel. Scale bar for 5× 500 μm and for 20× 100 μm (*P* = *, *n* = 12 for WT and 5 for *Gpr4* ^−/−^). (D, F) Normal distribution (Shapiro–Wilk test), unpaired *t*‐test. (A, C) Non‐parametric distribution (Shapiro–Wilk test), Mann–Whitney test, Error bars indicate (A) ± SEM or (B–F) ± SD. *P*‐values and *n* as indicated. AOM, azoxymethane, DSS, dextran sodium sulphate; WT, wild‐type.

**Fig. S3.** Signs of tumor development in progress colonoscopy and evidence of tumor formation upon AOM DSS colitis. (A) Progress colonoscopy and H&E. Scale bar for 5× 500 μm and for 10× 100 μm. (B) qPCR, *Bcl2* (*P* = *, *n* = 30 for WT and 14 for *Gpr4* ^−/−^). (C) IHC, Ki67. Scale bar 100 μm (*P* = ***, *n* = 8 each). (D) IHC, CXCL12. Scale bar for 5× 500 μm and for 20× 100 μm. Normal distribution (Shapiro–Wilk test), unpaired *t*‐test. Error bars indicate SD. *P*‐values and *n* as indicated. AOM, azoxymethane; DAB, 3,3′‐diaminobenzidine; DSS, dextran sodium sulphate; WT, wild‐type, immunohistochemistry.

**Fig. S4.** Determination of AP sites. Standard curve. AP, apurinic/apyrimidinic.

**Table S1.** Antibody panels used for flow cytometry. Antibodies used for FACS (M = mouse; R = rat; H = hamster; NA = not applicable).

## Data Availability

Supporting data are available as Supplementary Information and in a public database https://figshare.com/account/home#/projects/242957. The data underlying this article are available in a repository provided by the University of Zurich (the link to the repository still needs to be defined).
