# Supplementary figures and images for: Loss of proton‐sensing GPR4 reduces tumor progression in mouse models of colon cancer
Source: Mol Oncol. 2025 May 21;19(8):2196–211. doi: 10.1002/1878-0261.70045 (PMC12330923; doi:10.1002/1878-0261.70045)

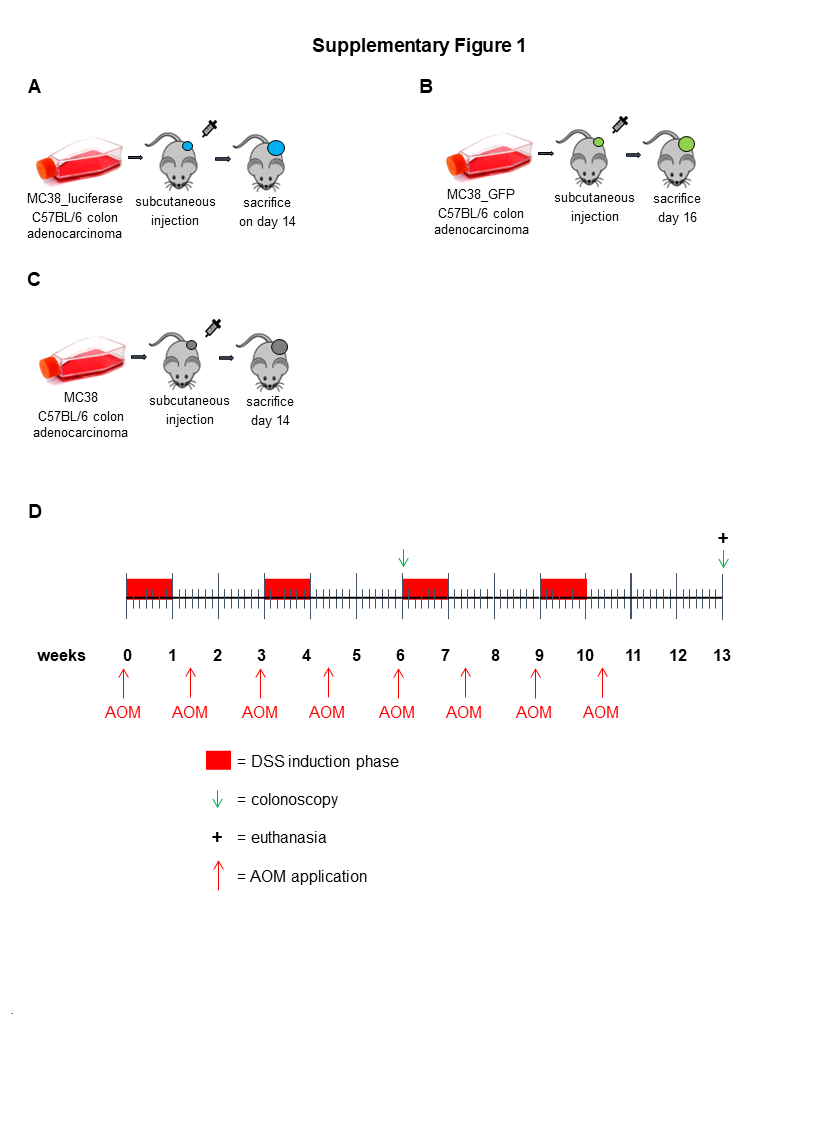

Supplement: Supplementary file 1 — Fig. S1. Experimental setup. 300 000 MC38 tumor cells expressing (A) luciferase, (B) GFP, and (C) unmodified cells were injected s.c. into WT and Gpr4 −/−. (D) Tumor induction with AOM/DSS. AOM, azoxymethane, DSS, dextran sodium sulphate; s.c., subcutaneous; WT, wild‐type. [file MOL2-19-2196-s001.tif]

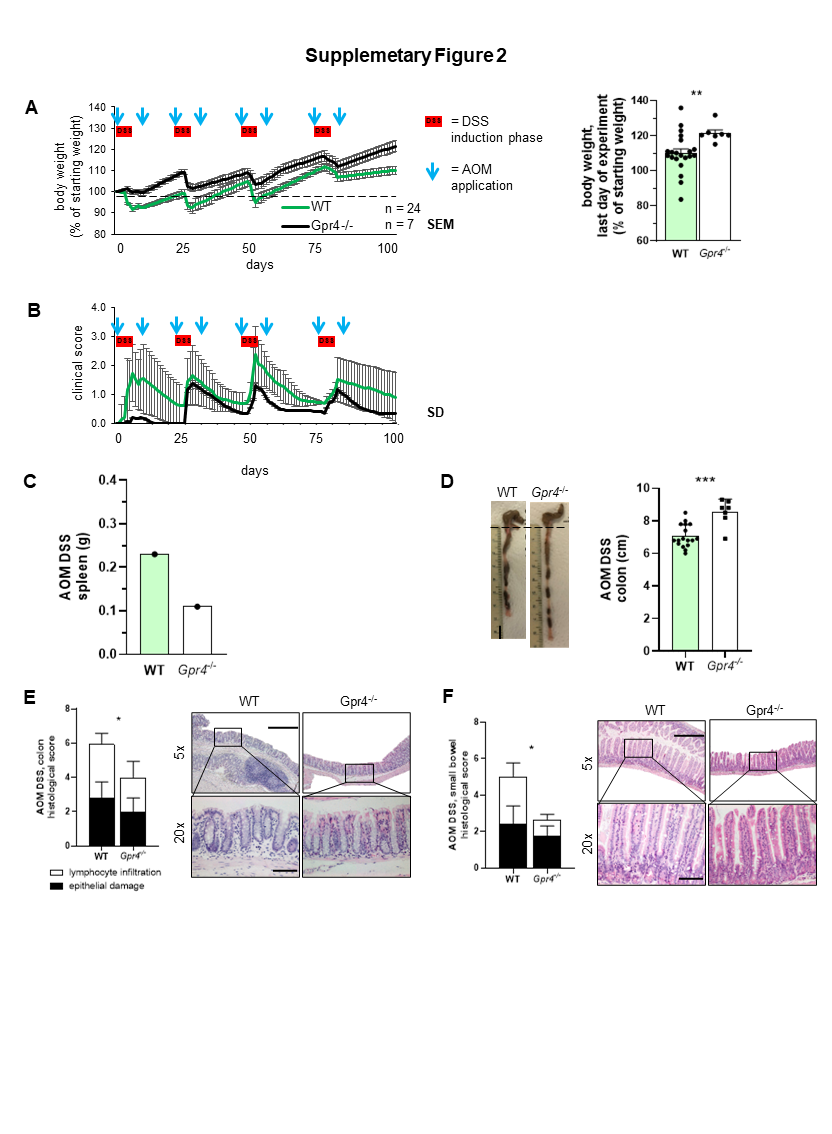

Supplement: Supplementary file 2 — Fig. S2. Decreased inflammation in Gpr4 −/− compared with WT mice upon AOM DSS colitis. (A) Body weight (P = **, n = 24 and 7). (B) Clinical score (n = 24 for WT and 7 for Gpr4 −/−). (C) Spleen weight (n = 1 each). (D) Colon length. Scale bar 10 mm (P = ***, n = 17 for WT and 7 for Gpr4 −/−). (E) Histological score, colon. Scale bar for 5 × 500 μm and for 20× 100 μm (P = *, n = 11 for WT and 6 for Gpr4 −/−). (F) Histological score, small bowel. Scale bar for 5× 500 μm and for 20× 100 μm (P = *, n = 12 for WT and 5 for Gpr4 −/−). (D, F) Normal distribution (Shapiro–Wilk test), unpaired t‐test. (A, C) Non‐parametric distribution (Shapiro–Wilk test), Mann–Whitney test, Error bars indicate (A) ± SEM or (B–F) ± SD. P‐values and n as indicated. AOM, azoxymethane, DSS, dextran sodium sulphate; WT, wild‐type. [file MOL2-19-2196-s004.tif]

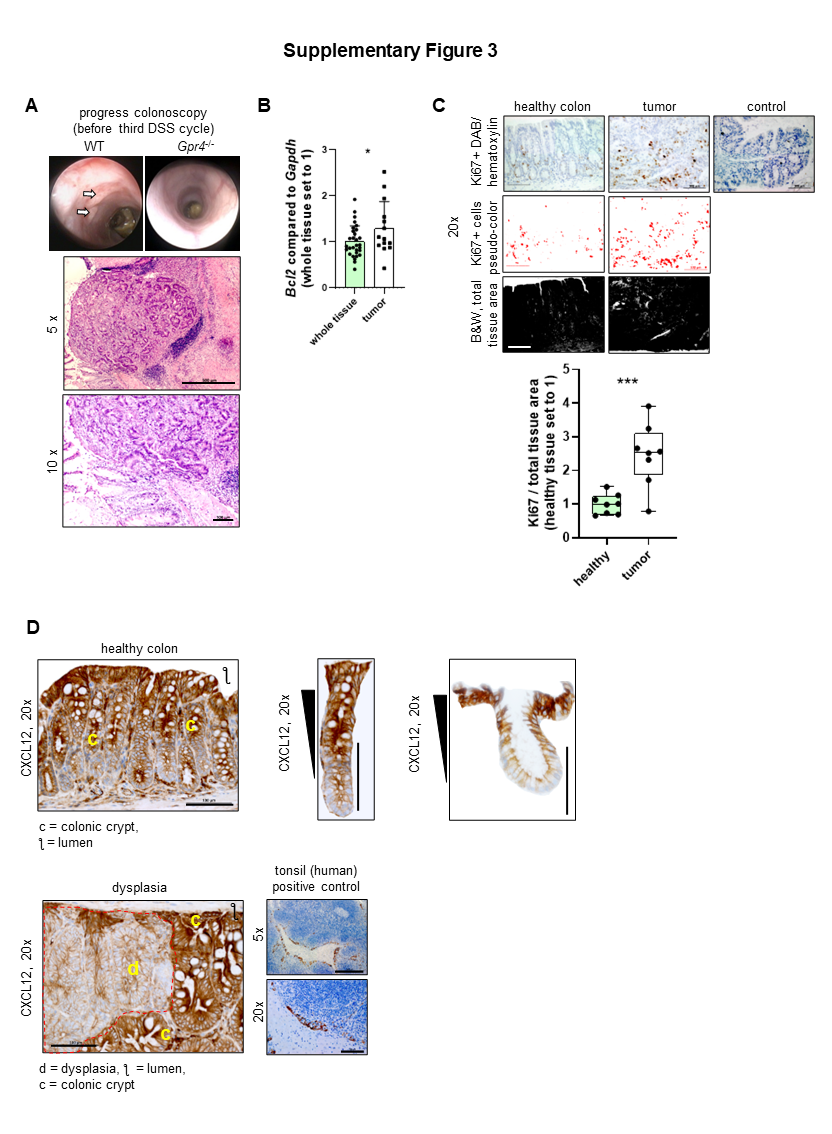

Supplement: Supplementary file 3 — Fig. S3. Signs of tumor development in progress colonoscopy and evidence of tumor formation upon AOM DSS colitis. (A) Progress colonoscopy and H&E. Scale bar for 5× 500 μm and for 10× 100 μm. (B) qPCR, Bcl2 (P = *, n = 30 for WT and 14 for Gpr4 −/−). (C) IHC, Ki67. Scale bar 100 μm (P = ***, n = 8 each). (D) IHC, CXCL12. Scale bar for 5× 500 μm and for 20× 100 μm. Normal distribution (Shapiro–Wilk test), unpaired t‐test. Error bars indicate SD. P‐values and n as indicated. AOM, azoxymethane; DAB, 3,3′‐diaminobenzidine; DSS, dextran sodium sulphate; WT, wild‐type, immunohistochemistry. [file MOL2-19-2196-s003.tif]

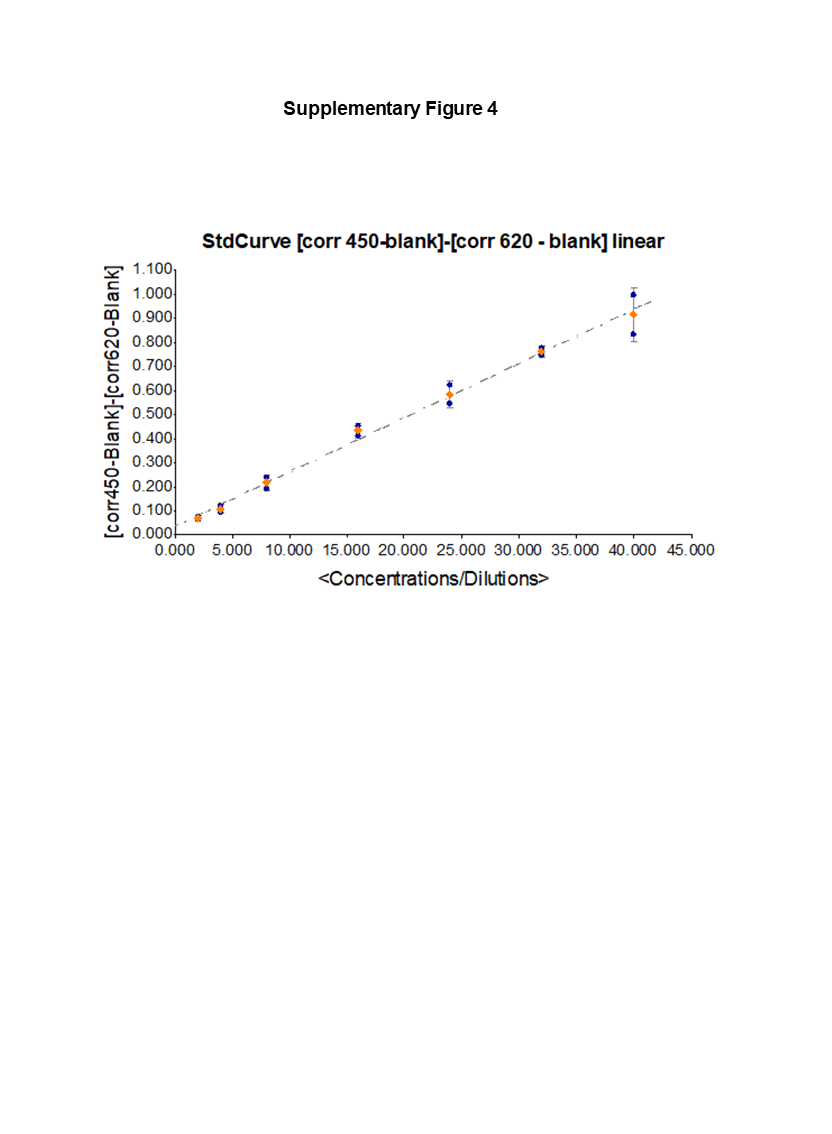

Supplement: Supplementary file 4 — Fig. S4. Determination of AP sites. Standard curve. AP, apurinic/apyrimidinic. [file MOL2-19-2196-s005.tif]
