# Supplementary material for: Loss of proton‐sensing GPR4 reduces tumor progression in mouse models of colon cancer
Source: Mol Oncol. 2025 May 21;19(8):2196–211. doi: 10.1002/1878-0261.70045 (PMC12330923; doi:10.1002/1878-0261.70045)
Supplement: Supplementary file 5 — Table S1. Antibody panels used for flow cytometry. Antibodies used for FACS (M = mouse; R = rat; H = hamster; NA = not applicable). [file MOL2-19-2196-s002.docx]

**SUPPLEMENTARY TABLE**

**Antibody panels used for flow cytometry**

| Panel | Fluorochrome channel | Target | Manufacturer | Reference number | Dilution | Host |
| --- | --- | --- | --- | --- | --- | --- |
| 1 | APC-Cy7 | viability marker | BioLegend | 423106 | 1:200 | NA |
| 1 | BV510, AmCyan | CD45 | BioLegend | 103138 | 1:400 | R |
| 1 | PE-Cy5 | B220 | Invitrogen | 15-0452-83 | 1:200 | R |
| 1 | BV785 | CD3 | BioLegend | 100232 | 1:200 | R |
| 1 | BV711 | NK1.1 | BioLegend | 108745 | 1:100 | M |
| 2 | APC-Cy7 | viability marker | BioLegend | 423106 | 1:200 | NA |
| 2 | BV510, AmCyan | CD45 | BioLegend | 103138 | 1:400 | R |
| 2 | PE-Cy5 | B220 | Invitrogen | 15-0452-83 | 1:200 | R |
| 2 | BV785 | CD3 | BioLegend | 100232 | 1:200 | R |
| 2 | BV605 | CD11b | BioLegend | 101257 | 1:200 | R |
| 2 | AF647 | Ly6G | BioLegend | 127609 | 1:200 | R |
| 2 | BV711 | Ly6C | BioLegend | 128037 | 1:200 | R |
| 2 | PE-Cy5.5 | MHC II | BioLegend | 107626 | 1:200 | R |
| 3 | APC-Cy7 | viability marker | BioLegend | 423106 | 1:200 | NA |
| 3 | BV650 | CD45 | BioLegend | 103151 | 1:200 | R |
| 3 | PE-Texas red | CD3 | BD | 562286 | 1:200 | H |
| 3 | PE-Cy5 | CD19 | Thermo Fisher | 15-0452-83 | 1:200 | R |
| 3 | BV510, AmCyan | Ly6G | BioLegend | 127633 | 1:200 | R |
| 3 | BV711 | Ly6C | BioLegend | 128037 | 1:200 | R |
| 3 | BV785 | NK1.1 | BioLegend | 108749 | 1:200 | M |
| 3 | AF700 | MHC II | BioLegend | 107622 | 1:200 | R |
| 3 | FITC | F4/80 | eBioscience | 11-4801-85 | 1:200 | R |

**Supplementary table 1:** Antibodies used for FACS (M = mouse; R = rat; H = hamster; NA = not applicable).
